# Serum YKL-40 as a Potential Biomarker for Sepsis in Term Neonates—A Pilot Study

**DOI:** 10.3390/children10050772

**Published:** 2023-04-25

**Authors:** Evangelia Steletou, Dimitra Metallinou, Alexandra Margeli, Theodoros Giannouchos, Athanasios Michos, Christina Kanaka-Gantenbein, Ioannis Papassotiriou, Tania Siahanidou

**Affiliations:** 1 Master of Science Program “Pediatric Infectious Diseases”, School of Medicine, National and Kapodistrian University of Athens, 11527 Athens, Greece; lia.stele@gmail.com; 2 Department of Midwifery, University of West Attica, 12243 Athens, Greece; metallinoudimitra@gmail.com; 3 Department of Clinical Biochemistry, “Aghia Sophia” Children’s Hospital, 11527 Athens, Greece; a.margeli@paidon-agiasofia.gr; 4 Department of Health Services Policy & Management, Arnold School of Public Health, University of South Carolina, Columbia, SC 29150, USA; giannout@mailbox.sc.edu; 5 First Department of Pediatrics, School of Medicine, National and Kapodistrian University of Athens, “Aghia Sophia” Children’s Hospital, 11527 Athens, Greece; amichos@med.uoa.gr (A.M.); chriskan@med.uoa.gr (C.K.-G.); ipapassotiriou@gmail.com (I.P.); 6 IFCC Emerging Technologies Division, Emerging Technologies in Pediatric Laboratory Medicine (C-ETPLM), 20159 Milano, Italy

**Keywords:** serum YKL-40, chitinase-3-like-1 protein, neonatal sepsis, biomarker, neonates, infants

## Abstract

Although YKL-40 is a promising diagnostic biomarker of sepsis in adults, its value in neonatal sepsis is not known. The study objectives included assessing the levels and diagnostic value of serum YKL-40 in term neonates with sepsis and comparing YKL-40 with other commonly used inflammatory biomarkers. In this pilot case–control study, 45 term neonates (30 septic and 15 non-septic, as controls), 4 to 28 days old, were prospectively studied. The International Pediatric Sepsis Consensus Conference criteria were applied to diagnose sepsis. During the acute phase (admission) and remission of sepsis, blood samples were collected from cases (while from controls they were only collected once) for routine laboratory tests, cultures, and the measurement of serum YKL-40 levels via Elisa. In the acute phase of sepsis, YKL-40 levels were significantly elevated in comparison with remission (*p* = 0.004) and controls (*p* = 0.003). YKL-40 levels did not differ significantly between patients in remission and controls (*p* = 0.431). Upon admission, YKL-40 levels correlated positively with white blood count, absolute neutrophil count, and CRP levels. Via ROC analysis, it was shown that YKL-40 levels upon admission were a significant indicator of sepsis (AUC = 0.771; 95% CI 0.632–0.911; *p* = 0.003). Serum YKL-40 might be considered as an adjuvant biomarker of sepsis in term neonates.

## 1. Introduction

In spite of advancements in diagnostic and treatment tools, neonatal sepsis, associated with an immature immune system, continues to contribute significantly to the global rates of neonatal morbidity and mortality [1,2,3]. Although there is currently no widespread agreement on the terms and standards for diagnosing neonatal sepsis, recent evidence has shown that neonates exhibit the highest prevalence of sepsis compared to all other age groups, with up to 22 cases per 1000 neonates, an increased mortality rate, and long-term neurological adverse outcomes [2,4,5]. The burden of neonatal sepsis highlights the need for a universal, clear, and prompt diagnosis of neonatal sepsis to avoid the overuse of antibiotics and to prevent severe complications [6].

The accurate diagnosis and prognosis of neonatal sepsis remains a scientific challenge and many potentially useful early biomarkers have been explored during recent years [7], including procalcitonin (Pct) [8,9,10,11,12], interleukin (IL)-6 and IL-8 [13,14,15,16], presepsin [17,18], serum amyloid A (SAA) [19], soluble triggering receptor expressed on myeloid cell-1 (TREM-1] [20], and many others [6,7]. Clinicians in neonatal intensive care units (NICUs) should have available biomarkers with both high sensitivity and specificity [6,7]. However, since none of the existing biomarkers are considered to be ideal [6,7], the investigation of new biomarkers of neonatal sepsis is rational.

Despite the large-scale research into biomarkers’ utility, there is no data available regarding the value of YKL-40 as a biomarker for neonatal sepsis. YKL-40, alias Chitinase-3-like protein 1 (CHI3L1) or glycoprotein-39, is a glycoprotein expressed in different types of cells that belongs to the family of chitinases [21]. The name YKL-40 was given due to the three N-terminal amino acids, namely tyrosine (Y), lysine (K), and leucine (L), and its molecular weight of 40 kDa [22]. The native protein’s crystal structure reveals that YKL-40 is comprised of a (β/α)8-barrel fold with a β + α domain insertion [23,24], which are necessary for the functions of YKL-40 in pathological and physiological processes. Although the biological role of YKL-40 remains partially understood, the model of its expression has been related to the activation of inflammatory responses (as being part of Th2-induced inflammation) and to pathogenic mechanisms that participate in processes such as tissue remodeling, asthma, fibrosis, and even carcinogenesis [22,25,26]. By controlling a number of vital biological processes such as apoptosis, pyroptosis, oxidant injury, inflammasome activation, inflammatory balance between cytokines Th1 and Th2, M2 macrophage differentiation, transforming growth factor beta 1 expression, dendritic cell accumulation, extracellular matrix regulation, and parenchymal scarring, YKL-40 plays critical roles in protecting against infectious agents, antigen- and oxidant-induced injury responses, and inflammation [22]. It is mainly expressed by neutrophils and activated macrophages and it is released from specific granules of neutrophils at the sites of inflammation [27]. YKL-40 has the ability to promote cell survival in challenging microenvironments, and increases rapidly in plasma in response to acute injury, inflammation, and infection. It has been reported that following endotoxin administration in humans, YKL-40 blood levels increase within 2 h and peak at 24 h [27]. Additionally, YKL-40 has been shown to enhance host tolerance while promoting pathogen clearance [28,29]. Nevertheless, this protective response, which is potentially beneficial during the acute phase of sepsis, can sometimes result in an unresolved, continual state of chronic inflammation without effectively clearing the pathogens. Taking all of these into consideration, YKL-40 might be a promising diagnostic and prognostic biomarker for many pathologies, including infections and inflammation [22,30].

As far as inflammation and infection are concerned, previous evidence has shown that high levels of serum YKL-40 are linked to acute or chronic inflammatory diseases [31], community-acquired pneumonia in adults [32,33], meningitis [34], and sepsis in adults [35]. More specifically, YKL-40 levels have been found to be higher in septic adults in comparison with healthy individuals and also higher in patients with positive blood cultures in comparison with septic patients who had negative blood cultures [35]. In addition, a positive correlation between the serum levels of YKL-40 and an already-known cytokine, IL-6, was shown [35]. According to another study, the severity of sepsis and mortality was associated with YKL-40 levels in septic adults [36]. Furthermore, in a prospective study in more than 90,000 Danish adults, elevated circulating YKL-40 levels were recognized as being a strong marker of an increased risk of future infectious diseases [37]. However, less is known about the association of YKL-40 and various outcomes in children. Only one study has been conducted in children with sepsis, including neonates as well, and it found a strong correlation between YKL-40 levels in blood and the severity of sepsis, but the number of neonates was very small (only four infants) [38].

In this study, we sought to determine the diagnostic value of serum YKL-40 levels in term neonates with sepsis and to compare YKL-40 with other indices of inflammation, such as C-reactive protein (CRP), white blood cell count (WBC), absolute neutrophil count (ANC), and platelet count (PLT), currently used in clinical practice as sepsis biomarkers.

## 2. Materials and Methods

### 2.1. Sample

In this pilot case–control study, term neonates, aged between 4 and 28 days old, who were admitted to the Level II (special care) neonatal unit of the First Department of Pediatrics, “Aghia Sophia” Children’s Hospital, during the period of 15 January 2020–15 January 2022, constituted the study sample. According to the hospital’s policy, all sick infants, up to 28 days of life, coming from home or referred from other hospitals, and not requiring intensive care, are admitted to this special care unit. Neonates with clinical signs and symptoms of sepsis [39] were allocated to the case group, whereas neonates of similar gestational age and birth weight, admitted to the unit for reasons other than sepsis (i.e., transient tachypnea, jaundice, regurgitation), were selected randomly to form the control group. The exclusion criteria were as follows: (1) prematurity, (2) congenital anomalies, (3) perinatal asphyxia, (4) history of surgery, (5) previous antibiotic treatment, and (6) age less than or equal to 3 days old.

Upon approval of the study by the hospital ethics committee, informed consent was also obtained from each neonate’s parent/caregiver.

### 2.2. Data Collection—Procedure

Sociodemographic and clinical characteristics of all neonates, such as age, birth weight, gestational age, gender, group of sepsis (culture-positive or culture-negative), and the microorganism that was grown on cultures, were recorded. Data from all participants were anonymized/deidentified through the creation of automatic coding by the database used.

According to the unit’s protocol, routine laboratory investigations included complete blood count (CBC), blood biochemistry, and serum CRP levels from peripheral vessels upon admission. The residual serum was aliquoted and stored at −80 °C until YKL-40 levels were assayed. Five hundred µL of blood collected in microvials was adequate for all analyses.

Moreover, analyses and cultures were performed on all septic neonates via blood, cerebrospinal fluid (CSF), and suprapubic (or catheter) urine sampling upon admission (Day 1), before the initiation of antibiotic treatment. Conventional culture methods were used for microbial detection and identification. Blood cultures were evaluated as being positive when only one microorganism was grown. Common commensal organisms such as coagulase-negative staphylococci, including *S. epidermidis*, were considered to be pathogenic when identified via culture in two or more blood specimens collected on separate occasions. The urine cultures were deemed positive only when one microorganism was grown at a concentration greater than 50.000 CFU/mL on a sample collected via suprapubic aspiration or urinary catheterization. The culture-positive group consisted of neonates with proven infection by culture(s), while the culture-negative group consisted of neonates fulfilling the sepsis criteria [39] but with negative cultures. Furthermore, a repeat blood sample was obtained on Days 7 to 10, after the end of antibiotic treatment and the clinical improvement in patients, with the goal of measuring the CRP and YKL-40 levels in sepsis remission. Blood was obtained only once from the control neonates.

YKL-40 concentrations were determined by means of an immunoenzymatic technique (DC3L10, Human Chitinase 3-like 1 Protein, R&D Systems, Minneapolis, MN, USA), pursuant to the manufacturer’s instructions. The immunoassay was designed to measure Chitinase 3-like 1 Protein in cell culture supernates, serum, plasma, or urine by utilizing an antibody specific for Chitinase 3-like 1 Protein with <0.5% cross-reactivity with available related molecules. A small amount of 10 µL of the sample was required for YKL-40 determination. The intraassay coefficient of variation (CV) was 4.3% to 4.7%, the interassay CV was 5.3% to 6.9%, and the sensitivity limit was 8.15 pg/mL.

### 2.3. Definitions

Systemic inflammatory response syndrome (SIRS) was defined based on a set of criteria established by the International Pediatric Sepsis Consensus Conference [39]. At least two of the four criteria relevant to (1) core temperature, (2) heart rate, (3) respiratory rate, or (4) leukocyte count should be fulfilled for SIRS definition; one of the two positive criteria should be abnormal temperature or leukocyte count. For sepsis determination, evidence of SIRS in the presence of or as a result of suspected (i.e., according to clinical findings) or proven infection (by culture, tissue stain, or PCR) was required [39].

### 2.4. Statistical Analysis

Since there exists no previous study on the literature surrounding circulating YKL-40 concentrations in neonates, we used data relevant to YKL-40 levels in peripheral blood in older healthy children [38] to calculate the sample size of the study. Assuming an alpha risk of 0.05, a power of 0.80, and a bilateral test, it was estimated that the minimum number of infants needed in each group to detect a significant difference of 1 standard deviation (SD) in mean YKL-40 levels between septic neonates and controls was 15 neonates.

A descriptive analysis was initially conducted to present the characteristics of all of the studied participants. Neonates were then stratified according to the diagnosis of sepsis (septic neonates or controls) and further according to culture positivity (culture-positive or culture-negative sepsis). For categorical variables, frequency was used, while for quantitative variables, mean and standard deviation or median and interquartile range (for variables not following a normal distribution) were used.

Bivariate analyses using t-test or Mann–Whitney test, as appropriate, for numerical variables, and Fisher’s exact test, for categorical variables, were conducted to examine statistically significant differences across all variables between septic neonates and controls. These included age, birth weight, gestational age, gender, and blood measurements of YKL-40, CRP, WBC, ANC, and PLT. Blood measurements were also compared between septic neonates with positive and negative culture(s). To compare YKL-40 and CRP levels between the acute and remission phases of the infection in septic neonates, the Wilcoxon signed rank test was used due to a non-normal distribution of these variables.

The correlation between YKL-40 levels and parameters of interest (CRP, WBC, ANC, and PLT) was evaluated based on Pearson or Spearman’s rho correlation coefficients, depending on the variables’ distributions. Finally, using the receiver operating characteristic (ROC) curve, we estimated the sensitivity and specificity of YKL-40 in neonates with sepsis to evaluate its diagnostic value and to propose a potential cut-off value. The statistical software IBM SPSS statistics version 26 (IBM Corporation, Somers, NY, USA) and Stata version 17.0 (StataCorp, College Station, TX, USA) were used for data analysis.

## 3. Results

### 3.1. Characteristics of Septic Neonates and Controls

In total, forty-five (n = 45) neonates constituted the study sample. Of them, 30 neonates fulfilled the criteria for sepsis, whereas 15 neonates composed the control group. The descriptive characteristics of cases and controls are presented extensively in Table 1. Overall, the majority of neonates were males, with an average age of 18.0 days, a gestational age of 38.4 weeks, and a birth weight of 3128 g. No significant differences were observed between septic and control neonates regarding age, gestational age, birth weight, or gender. Bacterial infections were confirmed via blood culture (n = 7) or urine culture (n = 13) in 20 out of 30 septic neonates (culture-positive group). The isolated bacteria in the culture-positive group were as follows: *Escherichia coli* (n = 11), *Enterococcus aerogenes* (n = 3), *Enterobacter aerogenes* (n = 3), *Staphylococcus aureus* (n = 2), and *Streptococcus agalactiae* (n = 1).

The summary of SIRS criteria in septic neonates is presented in Table 1. The number of infants fulfilling each of the SIRS criteria did not differ significantly between the culture-positive and the culture-negative septic groups. More specifically, abnormal core temperature was observed in all infants (*p* = 1.000), tachycardia or bradycardia in 8/20 vs. 7/10 (*p* = 0.245), increased respiratory rate in 13/20 vs. 8/10 (*p* = 0.675), and abnormal leukocyte count in 10/20 vs. 3/10 infants (*p* = 0.440) in the culture-positive and culture-negative septic group, respectively.

### 3.2. Inflammatory Markers and Serum YKL-40 Levels in Septic Neonates and Controls

Inflammatory markers (WBC, ANC, PLT, and CRP) and serum YKL-40 levels in the two study groups are depicted in Table 2. Upon admission, WBC, ANC, and CRP levels and serum YKL-40 concentrations were significantly higher in the septic neonates than in the controls (*p* = 0.017, *p* < 0.001, *p* < 0.001, and *p* = 0.003, respectively). Platelets were lower in the septic group in comparison with the control group, but the difference between groups did not reach statistical significance (*p* = 0.059).

In remission of sepsis (Days 7 to 10), serum YKL-40 (median (25th–75th): 24.1 (18.0–35.5) pg/mL) and CRP levels (median (25th–75th): 1.1 (0.40–1.9) mg/L) were significantly lower than levels upon admission (*p* = 0.004 and *p* < 0.001, respectively; Wilcoxon signed rank test). YKL-40 and CRP levels in remission of sepsis did not differ significantly between patients and controls (*p* = 0.431 and *p* = 0.174, respectively).

In septic neonates, no significant differences were observed upon admission between the culture-positive and the culture-negative group regarding YKL-40 levels (*p* = 0.061), WBC (*p* = 0.053), or PLT (*p* = 0.395). On the contrary, CRP levels and ANC were significantly higher in the culture-positive group than in the culture-negative group (*p* = 0.002 and *p* = 0.01, respectively) (Table 3).

### 3.3. Correlations between YKL-40 Levels and Inflammatory Markers upon Admission

**Figure 1:**
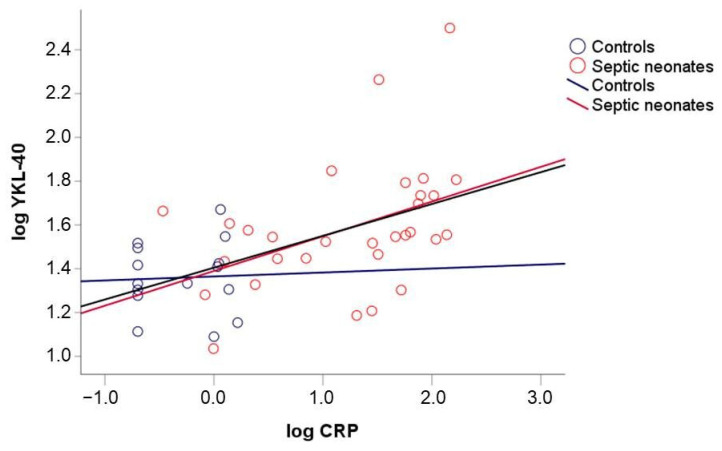
Correlation between YKL-40 and CRP levels overall (black line) in septic group (red line) and controls (blue line).

Significant correlations between serum YKL-40 levels upon admission and CRP (Figure 1), WBC, and ANC were observed in the total study population and in the group of septic neonates (Table 4). No significant correlation was found between YKL-40 levels and PLT. CRP levels also correlated significantly with WBC and ANC overall and in the septic group (Table 4). No significant correlation among YKL-40, CRP, WBC, ANC, and PLT was observed in the control group.

### 3.4. Diagnostic Value of YKL-40 Levels upon Admission

**Figure 2:**
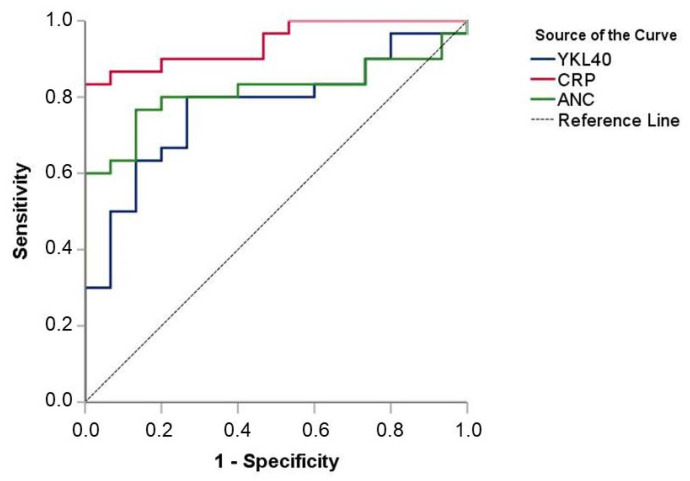
Diagnostic values of YKL-40, CRP, and ANC in neonatal sepsis (ROC curves).

As seen via ROC analysis, the YKL-40, CRP, and ANC values upon admission resulted in significant areas under the curve (AUC) for detecting sepsis: YKL-40 [AUC = 0.771 (95% CI 0.632–0.911), *p* = 0.003]; CRP [AUC = 0.942 (95% CI 0.879–1.000), *p* = 0.000]; and ANC [AUC = 0.816 (95% CI 0.690–0.941, *p* = 0.001]. The values of YKL-40 equal to or more than 26.8 pg/mL (cut-off point) had 80% sensitivity and 73% specificity for the detection of sepsis (Figure 2).

**Figure 3:**
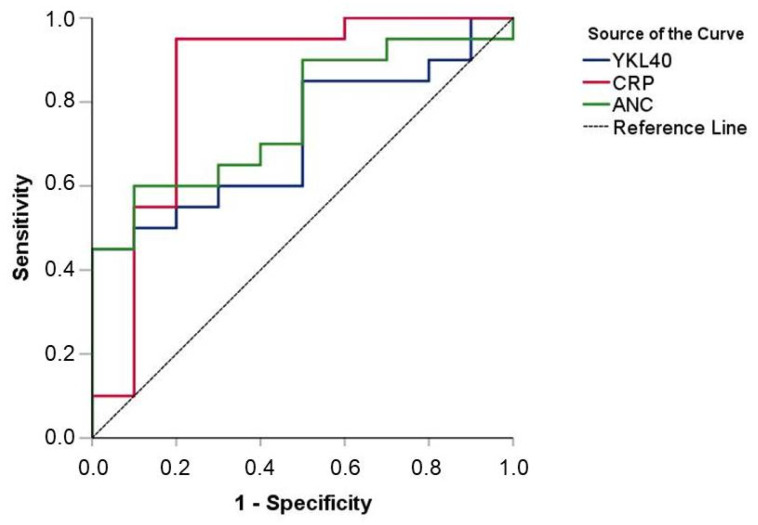
Diagnostic values of YKL-40, CRP, and ANC in discriminating between culture-positive and culture-negative sepsis (ROC curves).

The AUC for the diagnostic value of YKL-40 regarding the discrimination between culture-positive and culture-negative sepsis was 0.715 (95% CI 0.531–0.899) (*p* = 0.059). The ROC analysis for the diagnostic value of the CRP levels for the discrimination between culture-positive and culture-negative sepsis revealed significant results [AUC = 0.845 (95% CI 0.662–1.000), *p* = 0.002] which were better than those for ANC [AUC = 0.765 (95% CI 0.594–0.936), *p* = 0.02] (Figure 3).

## 4. Discussion

Numerous biomarkers have been explored regarding their diagnostic value for sepsis, but data on the efficacy of YKL-40 in neonates remain limited. The present study investigated the diagnostic value of serum YKL-40 levels in term neonates with sepsis and compared YKL-40 with other inflammatory biomarkers used in daily clinical practice. To the best of our knowledge, this is the first study to focus exclusively on neonates.

Previous studies have investigated YKL-40 levels in the pediatric population, yielding interesting and promising results. Kim et al. [40] examined the association between urinary YKL-40 levels and urinary tract infection (UTI) in 79 children with fever and an age range from 2 to 24 months. According to their results, urinary YKL-40 may be used in conjunction with standard analysis to identify a true UTI in feverish infants with false-positive or false-negative findings [40]. Similarly, a group of researchers from Egypt [41] concluded that the diagnostic value of urine YKL-40 was higher than that of urine neutrophil gelatinase-associated lipocalin (NGAL), proposing that it could be a valuable biomarker for the diagnosis of a UTI in febrile pediatric patients. More recently, Henckel et al. [42], in a cross-sectional study that included 29 10-year-old children who had been born preterm and were diagnosed with bronchopulmonary dysplasia, nominated YKL-40 as being a novel biomarker of altered lung development due to early-life inflammation in children with a history of prematurity. Furthermore, in children with juvenile idiopathic arthritis, YKL-40 was proposed as a marker of the efficacy of biologic treatment [43], whereas in children with human immunodeficiency virus (HIV) infection, YKL-40 levels were associated with viral load and poor virologic control, as well as immune dysregulation and microbial translocation [44]. Moreover, in children with cystic fibrosis, increased fecal YKL-40 levels have been reported and attributed to underlying intestinal inflammation [45]. Finally, earlier studies during childhood investigated YKL-40 in plasma as a prognostic factor for acute lymphoblastic leukemia [46], in the serum of children with hepatitis C and β-thalassemia as a marker of liver fibrosis, hepatic siderosis, and heart disease [47], and in the serum and bronchoalveolar lavage of children with community-acquired pneumonia [48].

In this pilot study, we demonstrated that serum YKL-40 levels were higher in septic neonates in the acute phase of sepsis (upon admission) in comparison with levels at remission of sepsis (Days 7–10) and levels in controls. YKL-40 could diagnose neonatal sepsis with a sensitivity of 80% and specificity of 73%, which are almost similar to, or even better than, those reported for Pct, showing a pooled sensitivity of 81% [10] and 85% [11] and specificity of 79% [10] and 54% [11] in meta-analyses. The sensitivity of YKL-40 for detecting neonatal sepsis is also similar to that of the cytokines IL-6 and IL-8 (79–83% and 78%, respectively) [14,15,16]. However, although the diagnostic value of YKL-40 in neonatal sepsis is substantial, it was found to be inferior to the diagnostic value of CRP in this study. Furthermore, CRP seems to be pivotal for the discrimination between culture-positive and culture-negative sepsis, while YKL-40 did not enable this discrimination in our pilot study sample. Interestingly, serum YKL-40 levels in the remission of sepsis, after completing the antibiotic treatment, did not differ significantly from the YKL-40 levels in controls. Consequently, our findings suggest that YKL-40, possibly in combination with other biomarkers, might constitute a clinically useful biomarker in septic neonates for monitoring the response to therapy and its effectiveness.

Hattori et al. [35] have already studied the diagnostic value of serum YKL-40 in adults and reported higher concentrations in septic patients than in controls, which is in good agreement with our findings. However, they also observed that YKL-40 levels were significantly higher in septic patients with positive cultures compared to the ones with negative cultures, whereas in our study this difference trended but did not reach the statistical significance level (*p* = 0.06). Moreover, the same research team proposed a cut-off value of YKL-40 that was potentially able to discriminate the septic shock subgroup from the severe sepsis subgroup, but not the patients from the controls. As far as other biomarkers were concerned, they presented a positive correlation between YKL-40 and IL-6 levels in the acute phase of sepsis [35]. Similarly, in our study, we found a positive correlation between YKL-40 levels and CRP, WBC, and ANC, biomarkers that are usually elevated in sepsis. This positive correlation between YKL-40 and WBC and ANC is primarily explained by the fact that YKL-40 is released from the granules of neutrophils [49].

Furthermore, our results are in line with previous findings in the literature, such as the ones by Liu et al. [38]. This team studied serum YKL-40 levels in 30 children diagnosed with severe sepsis; among them were also four newborns. Specifically, they demonstrated higher levels of YKL-40 in the septic group before treatment with continuous blood purification compared to the levels after treatment. The levels of YKL-40 after treatment were similar to those of the control group [38], which is similar to our results.

Our work is not without limitations, such as the fact that only surviving neonates were included in the study. In addition, as all neonates were hospitalized in a special care unit, none of the neonates in the experimental group presented septic shock or were so severely ill that they required intubation and mechanical ventilation. As a result, it was not possible to determine whether YKL-40 or any other of the evaluated biomarkers could predict adverse outcomes such as neonatal death or the need for respiratory support in an intensive care unit. Additionally, PCR panel screening methods were not used, and so we could not further study the causes of culture-negative cases of sepsis, i.e., bacteria not detected by cultures or viruses. A certain limitation of this pilot study is the small sample size. Although the study sample was rather adequate in number, according to sample size calculation, for the comparisons needed to be made between the case and the control group, it was not enough to conduct sub-group analyses, i.e., by types of microorganisms. Thus, a larger sample would be more favorable to fully clarify the properties of YKL-40 as a biomarker of neonatal sepsis. Another limitation of this study was the lack of Pct or IL-6 measurements, as both of them increase early, within 2 h and 2–4 h, respectively, after sepsis initiation [8,13], and IL-6 has been found to be positively correlated with YKL-40 levels in patients with sepsis [35]. Finally, no longitudinal blood samples were obtained so as to assess YKL-40 levels (e.g., every 24–48 h) after the initiation of antibiotic therapy with the objective of better investigating its kinetics and the association with clinical response.

## 5. Conclusions

Our pilot study confirms the effectiveness of YKL-40 as an early diagnostic biomarker of neonatal sepsis in term neonates, although YKL-40 levels did not differentiate between culture-positive and culture-negative sepsis in our study sample. It is critical, however, to conduct additional studies with larger sample sizes to clarify the optimal YKL-40 cut-off value for detecting sepsis and the potential of YKL-40 to subserve the clinical management of neonatal sepsis. A combination of appropriate biomarkers may lead to the development of a powerful model able to identify, diagnose, and monitor neonatal sepsis in a timely manner.

## Figures and Tables

**Table 1:** Descriptive characteristics of septic neonates and controls.

Variable	Overall (n = 45)	Septic (n = 30)	Controls (n = 15)	*p*-Value
Gender				0.660
Female	16 (35.6%)	10 (33.3%)	6 (40%)	
Male	29 (64.4%)	20 (66.7%)	9 (60%)	
Age (days)	18.0 (6.7)	17.9 (6.6)	18.1 (7.2)	0.549
Gestational age (weeks)	38.4 (1.2)	38.5 (1.2)	38.4 (1.3)	0.568
Birth weight (grams)	3,128 (363)	3,155 (303)	3,075 (467)	0.752
SIRS criteria				N/A
Core temperature of >38.5 °C or <36 °C		30 (100%)		
Tachycardia or bradycardia		15 (50%)		
Increased respiratory rate		21 (70%)		
Abnormal leukocyte count		13 (43.3%)		
Positive culture (blood, urine, or CSF)				N/A
No		10 (33.3%)		
Yes		20 (66.7%)		
Positive blood culture				N/A
No		23 (76.7%)		
Yes		7 (23.3%)		

Abbreviations: CSF, cerebrospinal fluid; N/A, not applicable.

**Table 2:** Inflammatory markers (WBC, ANC, PLT, and CRP) and serum YKL-40 levels in septic neonates upon admission and controls.

	Overall (n = 45)	Septic (n = 30)	Controls (n = 15)	*p*-Value *
Upon admission (Day 1)				
WBC (/mm^3^)				
Median (25th–75th)	11,030 (8660–15,940)	13,165 (8570–19,260)	10,250 (8660–11,030)	0.017
ANC (/mm^3^)				
Median (25th–75th)	3242 (1964–7970)	7089 (3192–11,388)	2010 (1426–2854)	<0.001
PLT (×10^3^/mm^3^)				
Median (25th–75th)	466 (369–609)	459 (361–555)	572 (418–702)	0.059
CRP (mg/L)				
Median (25th–75th)	3.4 (1–52.3)	30.3 (3.4–75)	0.6 (0.2–1.1)	<0.001
YKL-40 (pg/mL)				
Median (25th–75th)	32.9 (21.3–40.4)	35.5 (27.9–54.2)	21.5 (18.9–31.2)	0.003

* *p*-value for comparisons between septic neonates and controls; the Mann–Whitney test was used. Abbreviations: ANC, absolute neutrophil count; CRP, C-reactive protein; PLT, platelets; WBC, white blood cells.

**Table 3:** Inflammatory markers (WBC, ANC, PLT, and CRP) and serum YKL-40 levels upon admission in septic neonates stratified by culture positivity.

Upon Admission	Total Septic Group (n = 30)	Culture-Positive Group (n = 20)	Culture-Negative Group (n = 10)	*p*-Value
WBC (/mm^3^)				
Median (25th–75th)	13,165 (8570–19,260)	15,270 (10,690–20,225)	10,255 (6500–13,960)	0.053
ANC (/mm^3^)				
Median (25th–75th)	7089 (3192–11,388)	8674 (3,521–12,014)	3619 (1823–7199)	0.010
PLT (×10^3^/mm3)				
Median (25th–75th)	459 (361–555)	462 (350–580)	397 (363–510)	0.395
CRP (mg/L)				
Median (25th–75th)	30.3 (3.4–75)	54.7 (24.2–81.1)	2.2 (1–3.8)	0.002
YKL-40 (pg/mL)				
Median (25th–75th)	35.5 (27.9–54.2)	38.6 (31–63.1)	31.5 (21.3–35.9)	0.061

Abbreviations: ANC, absolute neutrophil count; CRP, C-reactive protein; PLT, platelets; WBC, white blood cells.

**Table 4:** Correlations between YKL-40 levels and CRP, WBC, ANC, and PLT upon admission.

	CRP	*p*-Value	YKL-40	*p*-Value
Overall (n = 45)				
YKL-40	0.59	<0.001	-	-
WBC	0.51	<0.001	0.39	0.009
ANC	0.61	<0.001	0.43	0.003
PLT	−0.08	0.599	−0.02	0.877
Septic group (n = 30)				
YKL-40	0.52	0.003	-	-
WBC	0.43	0.016	0.37	0.044
ANC	0.48	0.006	0.40	0.030
PLT	0.28	0.133	0.13	0.477

Abbreviations: ANC, absolute neutrophil count; CRP, C-reactive protein; PLT, platelets; WBC, white blood cells.

## Data Availability

The data presented in this study are available upon request from Tania Siahanidou (siahan@med.uoa.gr). The data are not publicly available due to privacy.
